# Knowledge, attitude and practice of diabetes among secondary school-going children in Bangladesh

**DOI:** 10.3389/fpubh.2022.1047617

**Published:** 2022-11-17

**Authors:** Farhana Akter, S. M. Mahbubur Rashid, Nazmul Alam, Nasrin Lipi, Md. Omar Qayum, Mehejabin Nurunnahar, Adnan Mannan

**Affiliations:** ^1^Department of Endocrinology, Chittagong Medical College, Chattogram, Bangladesh; ^2^Disease Biology & Molecular Epidemiology Research Group, Chattogram, Bangladesh; ^3^Department of Genetic Engineering & Biotechnology, University of Dhaka, Dhaka, Bangladesh; ^4^Department of Public Health, Asian University for Women, Chittagong, Bangladesh; ^5^Institute of Statistical Research and Training, University of Dhaka, Dhaka, Bangladesh; ^6^Department of Epidemiology, Institute of Epidemiology, Disease Control & Research (IEDCR), Dhaka, Bangladesh; ^7^Department of Genetic Engineering & Biotechnology, University of Chittagong, Chattogram, Bangladesh

**Keywords:** diabetes, school-going children, Bangladesh, awareness, healthy lifestyle

## Abstract

Diabetes Mellitus (DM) is a global public health concern. DM has been increasing alarmingly among the young people and childhood-onset has now become an emerging issue worldwide. Unlike other chronic diseases, DM requires constant and active attention of the patients, sometimes of their family members for successful management of this disease. Knowledge, attitude, and practices make significant differences at the population level, which largely depend on socioeconomic status, area of residence, level of education, and other socio-demographic attributes. A descriptive cross-sectional study was carried out among secondary school students in grades 6 through 10 attending schools in 18 districts of Bangladesh. A total of 2009 students were enrolled for the study from the selected schools and madrasas from Bengali, English, and Arabic medium. The majority of responders (79.34%) reported that they had heard of diabetes, however, only 45% knew that diabetes can cause blood glucose levels to rise. Among different therapeutic options, only 15% of students had heard the name of metformin, while 56.2% were familiar with insulin. English medium students were significantly more likely to have good knowledge than the Bangla medium students (19.31%, OR: 1. 44; 95% CI: 1.07, 1.95). Respondents of semi-urban (19.66%, OR: 1.7; 95% CI: 1.21, 2.36) and urban (18.47%, OR: 1.48; 95% CI: 1.17, 1.86) areas were more likely to have good knowledge than rural areas. Of the respondents, 20.61% did not know of any diabetic treatment options. Only a small percentage of students (11%) attended any diabetes education classes and 10.7% of students followed any recommended diabetes prevention methods. Knowledge, attitude, and practice related to diabetes were found to have been influenced by socioeconomic factors, societal practices, and behavioral patterns. This is the first nationwide research study in Bangladesh among secondary school students to study knowledge, attitudes, and practices related to diabetes. With a focus on Bangladesh's youthful population, this study sought to provide an informational framework that can be supportive for effective intervention to increase knowledge of diabetes and its implications.

## Introduction

Diabetes Mellitus (DM) is one of the major non-communicable diseases that has a significant association with increasing morbidity and mortality globally (1). According to the World Health Organization, DM is a silent global epidemic affecting mainly older adults; however, DM among the young population has been increasing consistently (1, 2). The prevalence of DM has been rising more rapidly in low- and middle-income countries than in high-income countries (1, 3, 4). Approximately 537 million adults (20–79 years) live with diabetes; and by 2045, this will rise to 783 million (1). The International Diabetes Federation estimated about 13.1 million people have diabetes in Bangladesh with a prevalence of 14.2% in adults and almost 5.7 million people have undetectable diabetes (5). The number is suspected to be doubled by 2045. Moreover, the prevalence of type-2 diabetes is increasing at an alarming rate among adults in both the rural and urban settings of the country (6, 7).

Besides adult Diabetes, childhood-onset DM has now become a burning issue worldwide (8–10). A recently published study conducted on US youths aged 10 to 19 showed that 22.3 and 13.8 per 100,000 youths were affected with Type 1 and Type 2 diabetes, respectively, in 2015 (8). The rates of incident diabetes cases have been increasing by about 1.9% for type 1 diabetes (T1DM) and 4.8% for type 2 diabetes (T2DM). Asian youths have shown the highest rate of increased prevalence of T2DM. The prevalence of impaired fasting glucose and type 2 diabetes among Bangladeshi youths aged 10–18 is 3.4 and 1.8%, respectively, which can be considered as very high (11).

Several factors have been attributed to the rising prevalence of diabetes including urbanization, adopting a western lifestyle, inadequate physical activity, and excess calorie intake (12). Both micro and macrovascular complications like cardiovascular diseases, retinopathy, nephropathy, and neuropathy have been known as long-term consequences of DM (13). This will significantly deteriorate patients' quality of life and creates a significant financial burden at the national and individual level. Prevention and self-care are the cornerstones of diabetes management as evidenced by various studies and populations (14–16). Unlike other chronic diseases, DM requires the constant, active participation of the patients, sometimes of their family members for successful management of this disease (15). Several studies have reported that knowledge, attitude, and practices at the population level that make significant differences largely depend on socioeconomic status, residing area, level of education, and other socio-demographic attributes (17, 18). Knowledge of diabetes mellitus at an early stage of life can assist in early diagnosis with subsequent minimizing of diabetes-related life-threatening complications (19–23). So, to prevent the upward trend of increasing young diabetic patients, it is necessary to know how much awareness has been built among people, especially children about different factors like lifestyle modification, dietary readjustment, treatment, medication adherence, physical activity, etc. Lack of appropriate perception about the disease results in poor compliance. However, this type of study has not been done yet on school-going children in Bangladesh. To build a strategy to create awareness, it is important to know to what extent the young people of this country are concerned about these health issues.

This is the first nationwide research study in Bangladesh among secondary school students to evaluate knowledge, attitudes, and practices related to diabetes. This study aimed to help guide effective intervention to raise awareness about diabetes and its consequences, especially among the young generation in Bangladesh, and contribute to much-needed healthy lifestyle adoption.

## Materials and methods

### Study design, participants and settings

Between January 2021 and January 2022, a descriptive cross-sectional study was carried out among high school students in grades 6 through 10 attending schools from Bengali, English and Arabic medium. For a better understanding of the real KAP scenarios of DM and geographic representation of Bangladesh, a total of 18 districts, drawn from all 8 administrative divisions of Bangladesh: Dhaka, Chattogram, Sylhet, Khulna, Rajshahi, Mymensingh, Barisal, and Rangpur were included in the study. A total of 18 districts were selected purposively, eight administrative districts of all divisions, top 7 densely populated districts to represent densely populated districts from census data and 3 indigenous districts were chosen to represent the indigenous population and hill tracts area. Using census data from the Bangladesh Bureau of Statistics (BBS) and nine of the education boards of Bangladesh, a total of 78 schools and madrasa were selected randomly from a list of all eligible and currently functioning schools. The schools and madrasas having the highest number of students from each district were selected. Selected schools follow both Bangla and English medium curricula while madrasas follow Arabic curriculum. English medium schools are mostly private, and students studying there mostly belong to the economically well-off families. A total of 2009 students were enrolled for the study from the selected schools and madrasas, ranging in grade level from sixth to tenth.

### Data collection procedure and tools

A pre-tested, structured close-ended questionnaire was used for data collection. The questionnaire consisted of five sections: socio-demographic characteristics; lifestyle-related practices; knowledge of diabetes, attitude toward diabetes, and practices on diabetes issues. The knowledge, attitude and practice level of the students were assessed based on the lifestyle modification/behavioral risk factors/choices of diabetes management included in the questionnaire. A semi-structured questionnaire was used to collect data by face-to-face interviews with trained interviewers having study background on life science or public health. This included family status, obesity, anxiety, salt intake, lack of exercise and physical activity, high fat diet, use of soft drinks, fast food consumption, symptoms of diabetes, and treatment of diabetes. The questionnaire was developed with input from study investigators with specialties in the field of epidemiology, endocrinology, public health and pediatrics. A pilot testing of the questionnaire was conducted among the children of Noakhali and Manikgonj district, the districts not included the main study. Feedback from pilot test has been incorporated for finalization of the questionnaire.

After informing and obtaining written permission from the school authorities and legal guardians, the purpose of the study was explained to the students. Parental assent was taken after explaining the study objectives and purpose of the study. The study tool was converted into the Bengali language for easy understanding of the questions. Anonymity and confidentiality were highly maintained. The collected data were entered into an excel spreadsheet.

### Statistical analysis

Survey data was imported through Microsoft Excel and analyzed via R version 3.5.2. Knowledge, attitude, and practice scores of the respondents were calculated from 15 knowledge questions, 6 attitude questions, and 9 practice questions. Scores were estimated using a score of 1 for each right answer and 0 for each wrong answer. Maximum score of knowledge, attitude and practice was 13, 6, and 8, respectively. Correct answer to 80% of questions is considered as the cutoff point to differentiate between good and poor knowledge, positive and negative attitude, good and bad practice (24).

Descriptive statistics were employed to describe socio demographic characteristics of the respondents and to demonstrate students' knowledge, attitude and practice toward diabetes. Pearson chi-square test was used to test the association of socio-demographic characteristics with the knowledge and practice status of the students. Multiple logistic regression analyses were done to estimate univariate and multivariable Odds ratio and 95% confidence interval to determine the predictors of knowledge level, where dependent variables were dichotomized as Good Knowledge and Poor Knowledge and predictor variables included socio-demographic variables including age, gender, medium of education, family income and father's and mother's education.

## Results

### Socio-demographics

In total, 2009 students took part in the survey; 970 of them (48.28%) were boys and 1,039 (51.71%) were girls. Bangla was the language of instruction for the majority of students (68.98%), followed by Madrasha (17.87%) and English (13.14). The survey covered 26.13% indigenous and 73% non-indigenous students. In comparison to rural (46.31%) and urban (41.76%) areas, a smaller share (11.89%) of the sample population came from semi-urban areas. Most study participants had a family income of ≤100 USD per month. In terms of their parents' education, the majority had only completed primary school (Table 1).

**Table 1:** Socio-demographic characteristics of the study participants (*N* = 2,009).

**Characteristics**		**Number of participants**	**Percentage (%)**
Gender	Male	970	48.28
	Female	1,039	51.71
Medium of education	Bangla	1,386	68.98
	English	264	13.14
	Madrasha	359	17.87
Place of residence	Rural	931	46.31
	Semi-Urban	239	11.89
	Urban	839	41.76
Ethnic origin	Indigenous	525	26.13
	Non-Indigenous	1,484	73.86
Father's education	None	162	8.06
	Primary	504	25.08
	Secondary	738	36.73
	Graduation	605	30.11
Mother's education	None	170	8.46
	Primary	576	28.67
	Secondary	794	39.52
	Graduation	469	23.34
Monthly family income (in taka)	≤10,000	485	24.14
	10,001–20,000	413	20.55
	20,001–30,000	460	22.89
	30,001–50,000	359	17.87
	≥50,001	292	14.53

### Knowledge

The majority of responders (79.34%) reported that they had heard of diabetes, however, only 45% knew that diabetes can cause blood glucose levels to rise. Only 15% of students had heard the name of metformin, while 56.2% were familiar with insulin. A whopping 58% of students believed that diabetes was a hereditary condition and 46.49% of students thought that it could reduce life expectancy (Table 2).

**Table 2:** Frequency distribution of the responses to knowledge questions.

**Questions**	**Response**	**Frequency**	**Percentage**
**Heard of diabetes**			
	Yes	1,594	79.34
	No	415	20.65
**What increases in blood when diabetes occurs?**			
	Glucose	911	45.34
	Other	422	21.00
	Don't Know	676	33.64
**Know about types of diabetes**			
	Yes	512	25.48
	No	1,497	74.51
**Diabetes increase the risk of other disease**			
	Yes	1,180	58.73
	No	829	41.27
**Diabetes, is a hereditary disease?**			
	Yes	1,164	57.93
	No	845	42.06
**Diabetes is a life -long disease**			
	Yes	782	38.92
	No	1,227	61.07
**Heard of Insulin?**			
	Yes	1,130	56.24
	No	879	43.75
**Diabetes reduces life expectancy**			
	Yes	934	46.49
	No	1,075	53.51
**Have you heard the name of “Metformin”?**			
	Yes	314	15.62
	No	1,695	84.37
**Symptoms of diabetes**			
	Frequent Urination	1,011	50.32
	Always feel tired	382	19.01
	Increasing weight	247	12.29
	Tired after meal	120	5.97
	Don't know	495	24.63
**Risk factors of diabetes**			
	Got it from family	552	27.47
	Eating Excess carbohydrates or sugary food	1,116	55.55
	Overweight	532	26.48
	Don't know	510	25.38

In multiple response analysis, 24.63% of respondents did not know any symptoms of diabetes, whereas 50.32% of respondents reported that frequent urination might be a sign of diabetes and 19.01% answered feeling tired all the time as a symptom. Regarding the risk factors for diabetes, 27.47% of respondents reported that the disease could occur from family heredity. Overconsumption of carbs or sugary meals can raise the risk of diabetes reported by 55.55% of the participants. Furthermore, being overweight was cited as a risk factor for diabetes by 26.48% of students, while 25.38% reported that they were unaware of any diabetes risk factors.

Overall, a higher proportion of girls were found to have good knowledge of diabetes than boys (18.57% vs. 14.53%). Interestingly, the difference in knowledge status is insignificant between mediums of education. Conversely, there were statistically significant differences in knowledge level by place of residence, ethnicity and monthly family income (Table 3).

**Table 3:** Frequency distribution of the responses to attitude questions.

**Questions**	**Response**	**Frequency**	**Percentage**
**Is it possible to control diabetes?**			
	Yes	558	27.77
	No	899	44.74
	Don't know	552	27.47
**What should a diabetic patient do to control diabetes?**			
	Balanced diet	190	9.45
	Exercise/Walking	599	29.81
	Both	897	44.64
	Don't know	323	16.07
**How can diabetes be treated?**			
	Regular exercise	1,167	58.08
	Balanced diet	404	20.11
	Regular checkup	329	16.37
	Insulin	857	42.65
	Metformin	269	13.39
	Paracetamol	88	4.38
	Don't know	414	20.61
**Long term use of Insulin intake increases complications**			
	Agree	234	11.64
	Disagree	342	17.02
	Don't know	1,433	71.33
**Controlled eating habits and regular exercise greatly reduce the risk of diabetes**			
	Agree	1,251	62.27
	Disagree	79	3.93
	Don't know	679	33.79

From the logistic regression analysis, we found that for 1 year change of age there were no significant changes in students' knowledge level regarding diabetes. English medium students were significantly more likely to have good knowledge than the Bangla medium students (19.31%, OR: 1. 44; CI: 1.07, 1.95). Respondent's place of residence was significantly associated with their knowledge regarding diabetes. Respondents of semi-urban (19.66%, OR: 1.7; CI: 1.21, 2.36) and urban (18.47%, OR: 1.48; CI: 1.17, 1.86) areas were more likely to have good knowledge than rural areas. Monthly family income level had a significant effect on students' knowledge. The likelihood of having good knowledge was more among students whose monthly family income was more than 500 USD. After adjusting for known covariates and confounding factors, we found no significant association between demographic characteristics and student knowledge about diabetes except the student's place of residence. Students of semi-urban areas were more likely to have good knowledge than those in rural areas (19.66%, OR: 1.62; CI: 1.14, 2.31) (Table 4).

**Table 4:** Frequency distribution of the responses to practice questions.

**Questions**	**Response**	**Frequency**	**Percentage**
**How often do you eat Fruit?**			
	Everyday	651	32.41
	More than twice in a week	649	32.31
	Once in a week	424	21.10
	Once in a month	254	12.64
	Never	31	1.54
**How often do you eat Vegetable?**			
	Everyday	1,260	62.72
	More than twice in a week	541	26.92
	Once in a week	139	6.91
	Once in a month	43	2.14
	Never	26	1.29
**How often do you eat Soft Drinks?**			
	Everyday	264	13.14
	More than twice in a week	267	13.29
	Once in a week	574	28.57
	Once in a month	694	34.54
	Never	210	10.45
**How often in a month do you eat junk/ fast food?**			
	≤10 days	1,667	82.97
	More than 10 days	342	17.02
**Have you ever attended an educational class on diabetes?**			
	Yes	221	11.00
	No	1,788	88.99
**Do you follow any measures to prevent diabetes?**			
	Yes	215	10.70
	No	1,225	60.97
	Don't know	569	28.32
**Do you follow any specific eating habits to avoid diabetic?**			
	Yes	652	32.45
	No	1,357	67.54
**Do you exercise/walk/run in a typical day?**			
	Yes	1,742	86.71
	No	218	10.85
**Do you play outdoor games in a day (cricket/football/others)?**			
	Yes	1,440	71.67
	No	569	28.32

### Attitude

All about 28% of respondents reported that it might be possible to control diabetes; 62.27% of them reported controlled diet and regular exercise significantly lower the chance of developing diabetes (Table 5). A frequent checkup is important for managing diabetes as reported by 16.37% of the students and 20.61% of students did not know of any diabetic treatment options. Of the participants, 42.65% reported that insulin could treat diabetes, while the majority of students (71.33%) were unaware that long-term insulin use could raise difficulties.

**Table 5:** Relationship among knowledge and practice with socio demographic variables.

**Variables**		**Knowledge**			**Practice**		
		**Good *n* (%)**	**Poor *n* (%)**	**χ2 (*p*-value)**	**Good *n* (%)**	**Poor *n* (%)**	**χ2 (*p*-value)**
**Gender**							
	Male	141 (14.53)	829 (85.46)	5.62 (0.017)	104 (10.87)	852 (86.95)	1.98 (−0.1591)
	Female	193 (18.57)	846 (81.41)		131 (13.04	873 (89.12	
**Medium of education**							
	Bangla	230 (16.59)	1,156 (83.41)	2.28 (−0.319)	146 (10.79)	1,206 (89.20)	5.97 (−0.0504)
	English	51 (19.31)	213 (80.68)		37 (14.12)	225 (85.87)	
	Madrasha	53 (14.76)	306 (85.24)		52 (15.02)	294 (84.97)	
**Place of residence**							
	Rural	132 (14.17)	799 (85.82)	7.68 (−0.022)	99 (10.93)	806 (89.06)	1.76 (−0.414)
	Semi-Urban	47 (19.66)	192 (80.33)		30 (12.82)	204 (87.17)	
	Urban	155 (18.47)	684 (81.53)		106 (12.91)	715 (87.08)	
**Father's education**							
	None	37 (24.46)	125 (77.16)	12.1 (−0.007)	19 (11.94)	140 (88.05)	1.16 (−0.763)
	Primary	81 (16.07)	423 (83.92)		55 (11.24)	434 (88.75)	
	Secondary	158 (21.41)	580 (78.59)		82 (11.53)	629 (88.46)	
	Graduation	148 (24.46)	457 (75.37)		79 (13.14)	522 (86.85)	
**Mother's education**							
	None	38 (22.35)	132 (77.64)	8.29 (−0.04)	19 (11.72)	143 (88.27)	3.7 (−0.295)
	Primary	101 (17.53)	475 (82.46)		64 (11.53)	491 (88.46)	
	Secondary	169 (21.28)	625 (78.71)		85 (10.89)	695 (89.10)	
	Graduation	116 (24.73)	353 (75.26)		67 (14.47)	396 (85.53)	
**Monthly family income**							
	≤10,000	61 (12.57)	424 (87.42)	13.17 (0.011)	50 (10.50)	426 (89.49)	1.75 (−0.781)
	10,001–20,000	65 (15.74)	348 (84.26)		49 (12.09)	356 (87.90)	
	20,001–30,000	82 (17.82)	378 (82.17)		58 (13.33)	377 (86.67)	
	30,001–50,000	61 (16.99)	298 (83.00)		43 (12.07)	313 (87.92)	
	≥50,001	65 (22.26)	227 (77.73)		35 (12.15)	253 (87.84)	

### Practice

To prevent diabetes, 32.41% of students reported that they eat fruits every day (Table 6). Most of the students reported doing physical activities daily (86.71%) and playing outdoor games (71.67%). Only a small percentage of students (11%) attended any diabetes education classes and 10.7% of students followed any recommended diabetes prevention methods.

**Table 6:** Results of multiple logistic regression on factors determining level of diabetes knowledge.

**Variables**		**Knowledge**			
		**Unadjusted**		**Adjusted**	
		**OR (95% CI)**	***P*-value**	**OR (95% CI)**	***P*-value**
Age		0.97 (0.92–1.02)	0.248	0.96 (0.91–1.01)	0.1747
Gender	Female	Ref		Ref	
	Male	0.82 (0.66–1.01)	0.0676	0.81 (0.64–1.00)	0.0553
Medium of education	Bangla	Ref		Ref	
	English	1.45 (1.07–1.95)	0.0156*	1.18 (0.81–1.70)	0.3808
	Madrasha	0.89 (0.66–1.19)	0.4242	0.91 (0.65–1.26)	0.5698
Place of residence	Rural	Ref		Ref	
	Semi-Urban	1.70 (1.21–2.36)	0.0018**	1.63 (1.14–2.31)	0.0067*
	Urban	1.48 (1.17–1.86)	0.00103**	1.25 (0.92–1.69)	0.1553
Monthly family income	<10,000	Ref		Ref	
	10,000–20,000	1.33 (0.95–1.86)	0.0923	0.79 (0.56–1.11)	0.1697
	20,000–30,000	1.21 (0.87–1.68)	0.253	1.18 (0.77–1.81)	0.4515
	30,000–50,000	1.34 (0.95–1.9)	0.0923	0.88 (0.63–1.23)	0.4514
	>50,000	1.89 (1.34–2.68)	<0.001***	0.88 (0.6–1.28)	0.5029
Father's education	None	Ref		Ref	
	Graduation	1.09 (0.73–1.67)	0.668	0.96 (0.51–1.83)	0.8934
	Primary	0.65 (0.42–1.01)	0.0508	0.72 (0.43–1.22)	0.2164
	Secondary	0.92 (0.62–1.4)	0.6891	1 (0.58–1.74)	0.9987
Mother's education	None	Ref		Ref	
	Graduation	1.14 (0.76–1.75)	0.534	0.8 (0.42–1.55)	0.5033
	Primary	0.74 (0.49–1.34)	0.157	0.89 (0.54–1.5)	0.665
	Secondary	0.94 (0.64–1.41)	0.758	0.85 (0.49–1.47)	0.549

* Significant with p < 0.05.

** Significant with p < 0.005.

*** Significant with p < 0.001.

Practices related to diabetes prevention were not found to be significantly different by gender, parents' educational levels, or place of living.

### Determinants of diabetes knowledge

It was found from logistic regression analysis that age was not a significant predictor of students' knowledge level regarding diabetes (Table 4). English medium students were significantly more likely to have good knowledge than the Bangla medium students (19.31%, OR: 1. 44; CI: 1.07, 1.95) as found from an unadjusted model; however, this finishing was not significant after adjustment with covariates. Respondent's place of residence was significantly associated with their knowledge regarding diabetes. Respondents from semi-urban areas (19.66%, OR: 1.7; CI: 1.21, 2.36) and urban (18.47%, OR: 1.48; CI: 1.17, 1.86) areas were more likely to have good knowledge than those living in rural areas. Monthly family income level had a significant effect on students' knowledge. The likelihood of having good knowledge was more among students whose monthly family income was more than USD 500, however, after adjusting for known covariates and confounding factors, we found no significant association between demographic characteristics and student knowledge about diabetes except for students' place of residence. Students of the semi-urban areas were more likely to have good knowledge than rural areas (Adjusted OR: 1.63; CI: 1.14–2.31).

## Discussion

This study aimed to study knowledge, attitudes and practices related to diabetes among secondary school students. Our findings revealed that a large proportion of students have overall poor knowledge and low awareness about diabetes, which is a major health issue of our time. This study included young population of diverse socio-demographic background to include indigenous students and others who opted for the private English medium schools. It was also highlighted that our school education systems rarely focus on heath literacy as it was found that only a small percentage of students' reporting having had attended session diabetes. Due to the rapid increase of diabetes cases worldwide; primary prevention, early diagnosis, and educational preventive measures are now being prioritized (13). It is suggested that the management of such health issues has been shown to be benefited greatly from people's education (17). Understanding diabetes and increasing general population awareness of the condition at a young age can help with early identification which ultimately can lessen life-threatening consequences that are linked to the disease (19, 20).

Overall participation rate was good in this study and the male-female ratio of the participants represents the current population scenario of Bangladesh (25). The study finds an average to low level of understanding of DM among young students in Bangladesh. While most of the participants knew about diabetes as a disease, but only half of them knew about the association of glucose and insulin with diabetes. Nearly half of the students were found to have awareness of the negative effect of taking excess carbohydrates in their meals, however, only one-fourth of the participants were able to recognize the association of obesity with diabetes which is very low compared to the findings of studies conducted on adolescents of Kuwait and Nigeria (12, 20). This finding might coincide with the fact that diabetes in the adult population in Bangladesh who are also obese or overweight has been reported to be high (26, 27).

In general, the school children exhibited poor knowledge of the signs and symptoms of diabetes, for example, only half of the participants knew excessive thirst and increased urination as a sign of diabetes. A similar knowledge pattern was observed in Nigerian adolescents but young students in Kuwait were found quite aware of the diabetes symptoms (12, 20). The fact that young students have such a limited understanding of the signs and symptoms of diabetes needs further attention, as early detection of the condition and fast treatment are both possible with early awareness of the symptoms (24–26).

It is rather concerning that nearly about three-fourths of participants were not aware that diabetes can be controlled through physical exercise, regulated diet intake and recommended medication including insulin when needed, which is very low in contrast with another study (23).

The study identified inadequate awareness programs running in the country that can contribute to raising awareness of diabetes and its associated management system, particularly among the young population in Bangladesh. Only 11% of the participants reported attending any educational programs on diabetes leaving a huge majority of the participants out of any kind of formal awareness-raising programs that can prevent diabetes in the future. However, the study found a satisfactory level of practices that ultimately can help in reducing the risk of diabetes in the future. About two-thirds of the participants used to have fruits in their meals regularly. Almost a universal level of the participants reported having vegetables in their meals at least twice a week.

Knowledge, attitude, and practice related to diabetes were found to have been influenced by socioeconomic factors, societal practices, and behavioral patterns (20). This study supports the mainstream findings since the regression model suggests that odds of having good knowledge about diabetes was significantly higher among students who belong to high-income families, those who live in urban or semi-urban areas, and studies in schools whose medium of instruction is English. However, this study did not find any significant differences in diabetes-related KAP status by their gender, medium of education, location, parents' education as well as family income.

This study was exclusive in that it focused on students in grades 6 through 10 who were between the ages of 9 and 16 and represented all geographical divisions of Bangladesh, encompassing rural, suburban, and urban areas. The study also includes students of indigenous origin. This is the first study in Bangladesh conducted to date among school children that evaluate the KAP of DM. The study also had a few limitations. Purposive sampling was used for selection of schools and the students as participants. Although the study area covered all eight administrative divisions in Bangladesh and study participants representing diverse socioeconomic status, however, precaution should be practiced for generalizability of the study finding. Data were collected through interview of school children without using particular scale, so collected data may suffer from recall bias or social desirability bias.

## Conclusion

The study finds an average to low level of knowledge and awareness of DM among secondary school students in Bangladesh, while most of them knew about diabetes itself but they lack some basic knowledge related to signs and symptoms, prevention and control measures. There was a substantial lack of accessible awareness-raising programs for the young population that may be attributable to overall poor KAP among school children. This study highly recommends initiating large-scale country-wide awareness-raising events where young-age people can participate and acquire knowledge and skills to shape up their plans in fighting chronic diseases like diabetes in the future.

## Data availability statement

The raw data supporting the conclusions of this article will be made available by the authors, without undue reservation.

## Ethics statement

The studies involving human participants were reviewed and approved by Ethical Review Committee (CMC/PG/2020/122) of Chittagong Medical College, Chattogram, Bangladesh. Written informed consent to participate in this study was provided by the participants' legal guardian/next of kin.

## Author contributions

FA, SR, MQ, NA, and AM conceived and designed the experiments. FA and AM coordinated the study. NL, MN, AM, and NA analyzed the data and drafted the manuscript. FA, MQ, MN, NA, and AM revised the manuscript. All authors contributed to the article and approved the submitted version.

## Conflict of interest

The authors declare that the research was conducted in the absence of any commercial or financial relationships that could be construed as a potential conflict of interest.

## Publisher's note

All claims expressed in this article are solely those of the authors and do not necessarily represent those of their affiliated organizations, or those of the publisher, the editors and the reviewers. Any product that may be evaluated in this article, or claim that may be made by its manufacturer, is not guaranteed or endorsed by the publisher.
